# Mohmmar Qadaffi, Open Access, and *Retrovirology*

**DOI:** 10.1186/1742-4690-1-24

**Published:** 2004-09-03

**Authors:** Kuan-Teh Jeang

**Affiliations:** 1 the National Institutes of Health, Bethesda, MD, USA

## Abstract

Retrovirology has been publishing as an Open Access online journal for approximately six months. In this editorial, I review the reasons for and the advantages of Open Access publishing, update our progress to date, and summarize where we intend to go with this journal.

## Qadaffi's lesson

Thirty-five years ago, on September 1^st^, 1969, I was an eleven year old boy living with my family in Benghazi, Libya. That morning, I awoke to an eerie silence. The normally bustling traffic outside of our apartment in a busy suburb of Benghazi was not to be heard. In fact, there was to be little or no traffic for the next several days. History will recount that day as the first day of Mohmmar Qadaffi's bloodless coup d'etat, seizing power in Libya.

What I learned that September week in 1969 was that access to and distribution of information tipped the fate of a state. On that September day, there were in fact two groups of army officers vying to overthrow the then enfeebled Libyan King Idris. Qadaffi, at the time only twenty-seven years old, headed a small group of very junior officers. He, however, had a bold and swift strategy. His first targets were not the dams, the power stations, the police stations...; but the radio stations (in 1969 television had a very minor penetrance in Libya) and newspapers. By dint of controlling the airwaves and printing presses and by freely distributing his information during time of chaos, Qadaffi held sway over the minds and the sentiments of the populace. He was thus able to pre-empt the efforts of a larger competing group of more senior army officers.

What does Qadaffi's story have to do with Open Access publishing? Imagine how the outcome might have been different in 1969 if broadcast radio was not freely and openly accessible by the masses but was an expensive subscription service limited to a few. In that setting, and perhaps in all settings, information that reaches a little audience at a steep cost is information with little effect.

## Retrovirology, freely and openly accessible by all

Curiously, the force that drives a coup d'etat is the same that motivates scientists to publish (although I am not suggesting that scientists are like Qadaffi). As scientists, we want everyone who is interested to read what we have done; and ideally we want that information to get to the widest audience in the fastest, most informative, and least expensive manner. Until now, ideal and reality were two entirely different matters. Traditional publishing in print journals reaches a limited audience, is slow, bulky, and expensive. By its very nature (i.e. pages of paper are heavy to mail and expensive to print), there is a real impetus on the part of print publishers to restrict the space available to authors. We are all too familiar with the results: abbreviated presentation of methods and text, the necessity to "inadvertently" not cite many colleagues' publications, and the ubiquitous use of microscopic figure panels that provoke blurred vision and headaches. I daresay that many after having read a short "letter" or a "brief definitive" report have been left scratching heads and wondering how the experiments were done, where did the vectors come from, and why many controls were definitely missing.

For the retrovirology community, *Retrovirology *aims to change such "reality". *Retrovirology *is published by BioMed Central, an independent publisher committed to ensuring immediate free access to peer-reviewed biomedical research. *Retrovirology *is Open Access which means that it is universally and freely available online to everyone. You as the author retain copyright, and your paper is guaranteed to be archived in at least one internationally recognised repository [1]. More importantly, publishing in *Retrovirology *is rapid. For example, a full research article was reviewed and published in less than 20 days [2]; and a review article was similarly processed in 8 days [3]. *Retrovirology *will also give you all the space and all the colored prints (at no extra charge) to present your findings informatively and attractively. If we don't publish your paper, we will tell you honestly what is scientifically deficient about your work. I promise not to obfuscate myself using annoyingly belittling mantra that my editorial decision was guided by "intense competition for space".

## Judge a book by its cover?

This is my second editorial for *Retrovirology *readers [4]. *Retrovirology *has been publishing on-line now for 6 months. While a first editorial can speak of dreams and aspirations, the burden of a second editorial is to demonstrate results and progress. Aided by an international cohort of extremely capable editors (Monsef Benkirane, Ben Berkhout, Masahiro Fujii, Michael Lairmore, Andrew Lever, and Mark Wainberg) and sixty editorial board members [5], I am indeed encouraged and gratified by *Retrovirology's *initial achievements. As of this writing, we have published 22 papers. These are good papers; and importantly, these papers are being widely-read. I can say with some pride that half (11 out of 22; see Table 1) of our published papers have been read more than 1,000 times each. The remaining papers are not far behind; our most recently published paper, barely 1 week on-line, has already had more than 200 readers. Given that retrovirology is a relatively small community of researchers, my personal experience of twenty years in this field tells me that these are very respectable numbers.

**Table 1:** Access statistics for the top 11 *Retrovirology *articles.

	**Article**	**Access Count**
1	**Early steps of retrovirus replicative cycle** Sébastien Nisole, Ali Saïb *Retrovirology *2004, **1**: 9	1890
2	**Establishment of a novel CCR5 and CXCR4 expressing CD4+ cell line which is highly sensitive to HIV and suitable for high-throughput evaluation of CCR5 and CXCR4 antagonists** Katrien Princen, Sigrid Hatse, Kurt Vermeire, Erik De Clercq, Dominique Schols *Retrovirology *2004, **1**: 2	1602
3	**Evolution of the HIV-1 envelope glycoproteins with a disulfide bond between gp120 and gp41** Rogier W Sanders, Martijn M Dankers, Els Busser, Michael Caffrey, John P Moore, Ben Berkhout *Retrovirology *2004, **1**: 3	1332
4	**Use of a multi-virus array for the study of human viral and retroviral pathogens: gene expression studies and ChIP-chip analysis** Elodie Ghedin, Anne Pumfery, Cynthia de la Fuente, Karen Yao, Naomi Miller, Vincent Lacoste, John Quackenbush, Steven Jacobson, Fatah Kashanchi *Retrovirology *2004, **1**: 10	1313
5	**Increased mortality associated with HTLV-II infection in blood donors: a prospective cohort study** Jennie R Orland, Baoguang Wang, David J Wright, Catharie C Nass, George Garratty, James W Smith, Bruce Newman, Donna M Smith, Edward L Murphy, *Retrovirology *2004, **1**: 4	1209
6	***Retrovirology *and young Turks... ** Kuan-Teh Jeang *Retrovirology *2004, **1**: 1	1140
7	**HIV-1 gene expression: lessons from provirus and non-integrated DNA** Yuntao Wu *Retrovirology *2004, **1**: 13	1103
8	**Multi-faceted, multi-versatile microarray: simultaneous detection of many viruses and their expression profiles** Biehuoy Shieh, Ching Li *Retrovirology *2004, **1**: 11	1097
9	**Two discrete events, human T-cell leukemia virus type I Tax oncoprotein expression and a separate stress stimulus, are required for induction of apoptosis in T-cells** Takefumi Kasai, Kuan-Teh Jeang *Retrovirology *2004, **1**: 7	1088
10	**Apoptosis of uninfected cells induced by HIV envelope glycoproteins** Barbara Ahr, Véronique Robert-Hebmann, Christian Devaux, Martine Biard-Piechaczyk *Retrovirology *2004, **1**: 12	1076
11	**HIV CTL escape: at what cost?** Stephen M Smith *Retrovirology *2004, **1**: 8	1022

Despite that many of you would argue to me that you would rather publish in *Cell*, *Science*, and *Nature *rather than *Retrovirology*. I wouldn't dispute you on that point. On the other hand, I would ask you to pause and think about a refrain that I am sure all of you have taught your children, "Don't judge a book by its cover!" Please, don't sell your own work short; and please don't belittle the objectivity of your colleagues. If you have a wonderful piece of work, do you truly need the cover of *Cell *to establish that point? On the other hand, if you have a horrible study, do you think that hiding it behind the cover of *Nature *would fool many of your colleagues for long? There was a time in the print age when display space on reading room shelves was limited and perhaps justifiably dominated by "the big three". Today, *Retrovirology *will get your paper listed in PubMed within 48 hours of acceptance for publication. Anyone in this electronic era whose reading habits are not guided by PubMed in one way or another is probably someone who you may not care to read your study. In short, *Retrovirology *will promise your immediacy of publication and visibility for your work. Your papers will be well-read by your colleagues. Beyond that, I believe, and I think you do too, the impact of your work should rest on its merits and not on our or any other journal's cover.

## Open Access is not free lunch

Besides teaching about book covers, I think the other concept that we teach our children is that "There is no free lunch!" At the end of the day, anything worth doing has a price tag. I, my colleague editors, and all the editorial board members at *Retrovirology *work for free for the journal. We can do this because we collect a salary elsewhere doing research. On the other hand, we do have a dedicated staff based at the publisher in London. No one would question that the salaries for these professionals as well as other publication costs must be borne. To fund these costs, from October 1, 2004, authors of articles accepted for publication will be asked to pay a single all-inclusive article-processing charge (APC) of US$600; there are no additional charges beyond this sum. *Retrovirology *will not levy additional page or colour charges on top of this fee. You are free to publish any number of colour figures and photographs, at no extra cost.

I support the need for APC, because without this *Retrovirology *cannot be published. I think most of you will agree based on your experience with other publishers that US$600 is more than modest and reasonable. As a point of comparison, the Public Library of Science has set up two new Open Access journals, and has set their APCs at US$1500, fully two and one-half times our charge, for each accepted article [6]. Nonetheless, I realize that any APC fee can present an unacceptable financial hardship for some authors. These authors should feel free to contact me in confidence, and I will consider fee-waiver requests on a case-by-case basis.

In closing, let me thank those of you who have read, submitted and reviewed papers for *Retrovirology*. More than anything else, *Retrovirology *is not my journal as much as it is your journal. I welcome your advice, and I look forward to hearing from you.

## Abbreviations

APC = article-processing charge.
